# A Retrospective Exploratory Analysis on Cardiovascular Risk and Cognitive Dysfunction in Multiple Sclerosis

**DOI:** 10.3390/brainsci11040502

**Published:** 2021-04-16

**Authors:** Antonio Reia, Martina Petruzzo, Fabrizia Falco, Teresa Costabile, Matteo Conenna, Antonio Carotenuto, Maria Petracca, Giuseppe Servillo, Roberta Lanzillo, Vincenzo Brescia Morra, Marcello Moccia

**Affiliations:** Department of Neurosciences, Reproductive Science and Odontostomatology, Federico II University, 80131 Naples, Italy; an.reia91@gmail.com (A.R.); martinapetruzzo@gmail.com (M.P.); fabriziafalco@yahoo.it (F.F.); teresa.costabile@gmail.com (T.C.); matteo.conenna@gmail.com (M.C.); carotenuto.antonio87@gmail.com (A.C.); maria@petraccas.it (M.P.); giuseppe.servillo@unina.it (G.S.); robertalanzillo@libero.it (R.L.); vincenzo.bresciamorra2@unina.it (V.B.M.)

**Keywords:** multiple sclerosis, cardiovascular, cognitive, verbal learning

## Abstract

Background. Cardiovascular comorbidities have been associated with cognitive decline in the general population. Objectives. To evaluate the associations between cardiovascular risk and neuropsychological performances in MS. Methods. This is a retrospective study, including 69 MS patients. For all patients, we calculated the Framingham risk score, which provides the 10-year probability of developing macrovascular disease, using age, sex, diabetes, smoking, systolic blood pressure, and cholesterol levels as input variables. Cognitive function was examined with the Brief International Cognitive Assessment for MS (BICAMS), including the Symbol Digit Modalities Test (SDMT), the California Verbal Learning Test-II (CVLT-II), and the Brief Visuospatial Memory Test-Revised (BVMT-R). Results. Each point increase of the Framingham risk score corresponded to 0.21 lower CVLT-II score. Looking at Framingham risk score components, male sex and higher total cholesterol levels corresponded to lower CVLT scores (Coeff = −8.54; 95%CI = −15.51, −1.57; and Coeff = −0.11; 95%CI = −0.20, −0.02, respectively). No associations were found between cardiovascular risk and SDMT or BVMT-R. Conclusions. In our exploratory analyses, cardiovascular risk was associated with verbal learning dysfunction in MS. Lifestyle and pharmacological interventions on cardiovascular risk factors should be considered carefully in the management of MS, given the possible effects on cognitive function.

## 1. Introduction

Multiple sclerosis (MS) is a chronic disease of the central nervous system and represents the most important cause of non-traumatic disability in young people [1]. The aetiology of MS is still uncertain; genetic and environmental factors have been equally suggested to be causative, but there is no definitive agreement about their role and interactions [2]. The burden of MS is mostly related to motor disability, though cognitive dysfunction can occur in up to 70% of patients and impacts on patients’ quality of life and functioning [1]. Cognitive impairment can affect all MS subtypes (relapsing and progressive) [3,4,5] and its prevalence is associated with longer disease duration, impaired brain networks [6], and accelerated brain atrophy [5,7]. Attention and information processing speed are the earliest and most impaired functions in MS [8], and can be assessed with the Symbol Digit Modalities Test (SDMT), that is considered the most useful test to assess global cognition in MS [9]. Memory, executive, and visuo-spatial deficits also occur [10], and, thus, neuropsychological batteries have been developed to quickly screen multiple cognitive domains in MS clinical practice.

Cardiovascular comorbidities have already been associated with poorer MS motor progression [11,12,13,14], and could also be involved in the development and worsening of specific cognitive dysfunction. Several studies in the general population showed that cardiovascular risk factors have a negative impact on neuropsychological performances [15,16,17,18], and, more in general, on brain integrity [19]. In this exploratory study we used the Framingham risk score to assess the global cardiovascular risk, represented as the percent risk of developing heart failure, coronary, cerebral or peripheral artery disease within 10 years, using age, sex, diabetes, smoking, systolic blood pressure, and cholesterol levels as input variables [20]. The Framingham risk score has already been associated with the 5-year risk of MS progression [21], and we hereby aim to assess its possible associations with cognitive impairment in MS.

## 2. Methods

### 2.1. Study Design and Population

This is a retrospective study, aiming to evaluate the possible association between the Framingham cardiovascular risk score (and its components) and neuropsychological performances in MS. The study was approved by the Federico II Ethics Committee (355/19). All patients signed informed consent authorizing the use of anonymized data collected routinely as part of the clinical practice, in line with data protection regulation (GDPR EU2016/679). The study was performed in accordance with good clinical practice and Declaration of Helsinki. Patients were recruited consecutively at the MS Clinical Care and Research Centre of the Federico II University of Naples, Italy, between January and February 2020.

Inclusion criteria were: (1) MS diagnosis [22]; (2) consent to neuropsychological assessment and collection of cardiovascular variables. Exclusion criteria were: (1) age <18 years; (2) participation in clinical trials.

### 2.2. Neuropsychological Variables

All patients underwent the Brief International Cognitive Assessment for MS (BICAMS) neuropsychological battery, which includes the following tests: the Symbol Digit Modalities Test (SDMT), evaluating attention and information processing speed; the California Verbal Learning Test-II (CVLT-II), evaluating memory and verbal learning; and the Brief Visuospatial Memory Test-Revised (BVMTR), evaluating visuo-spatial learning.

Results were corrected for age, sex, and education, according to the Italian normative values [23]. We then calculated the corresponding cerebral functional system (FS) score (0 corresponds to normal BICAMS tests, 1 corresponds to one impaired BICAMS test, ≥2 corresponds to at least 2 impaired BICAMS tests), as from previous studies [24].

### 2.3. Cardiovascular Variables

We calculated the Framingham risk score by assessing the following cardiovascular risk factors: age, sex, diabetes (yes/no), smoking (yes/no), systolic arterial blood pressure, and use of antihypertensive drugs, total and HDL cholesterol levels (using CHOL2 and HDLC4 kits, respectively, on a COBAS c501 analyzer, Roche Diagnostics, Switzerland). Further details on collection of cardiovascular variables are reported elsewhere [11].

### 2.4. MS Clinical Variables

MS disability was scored with the Expanded Disability Status Scale (EDSS) by certified physicians. Disease duration was calculated as the difference between the reported date of MS diagnosis and the neuropsychological evaluation.

### 2.5. Statistical Analysis

Results are presented as mean (standard deviation), number (percent), and median (range), as appropriate. For statistical purposes, we included neuropsychological scores adjusted for age, sex, and education. We preliminary evaluated the associations between the corrected neuropsychological scores and the demographic and MS clinical variables (age, sex, education, disease duration, EDSS), using linear regression models.

To achieve study objectives, we evaluated the associations between neuropsychological scores and the Framingham risk score using different linear regression models (one for each of the BICAMS tests). We included age, sex, and education as covariates; these covariates were selected since they are known to affect both cardiovascular risk and neuropsychological performance (i.e., elderly, male and less educated people have higher cardiovascular risk and worse neuropsychological performance). Then, we included the components of the Framingham risk score in different stepwise linear regression models (one for each of the BICAMS tests); we used *p* = 0.20 as critical value to select the best correlates among age, sex, diabetes, smoking, systolic blood pressure, use of antihypertensive treatments, HDL, and total cholesterol levels. Finally, we evaluated the association between cerebral functional system score and the Framingham risk score using a linear regression model.

Statistical analyses were run using Stata 15.0. Results are presented as coefficients (Coeff), 95% confidence intervals (95% CI). Normal distribution of residuals was checked using both statistical and graphical approaches.

## 3. Results

We enrolled 69 MS patients; demographic, clinical, cardiovascular, and neuropsychological variables are reported in Table 1.

We found that lower SDMT scores corresponded to longer disease duration (Coeff:-0.38; 95%CI: −0.71, −0.48) and higher EDSS (Coeff: −2.28; 95% CI: −3.75, −0.80); there were no associations between SDMT and demographic variables (age, sex, and education). The CVLT-II and BVMT-R were not associated with neither demographics nor MS clinical variables.

**Figure 1:**
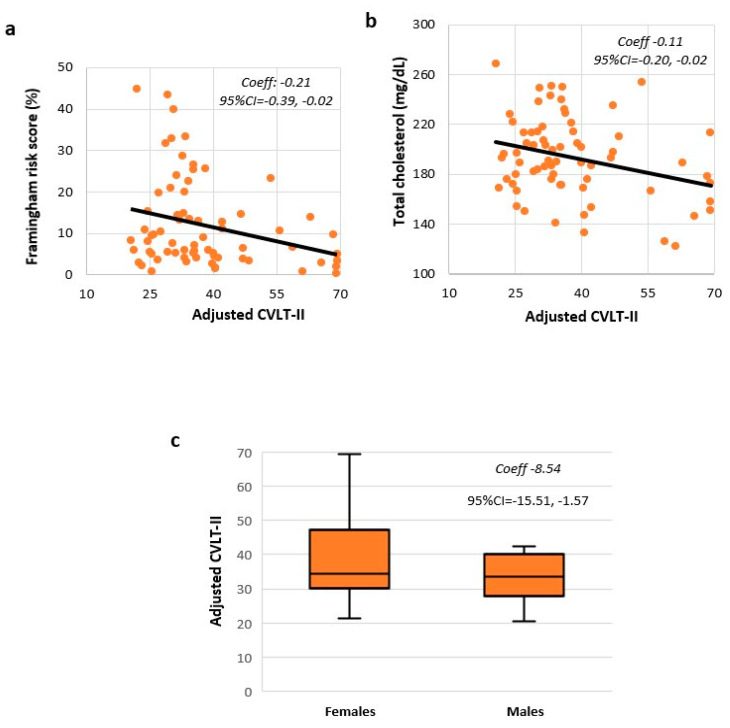
Cardiovascular risk and CVLT-II. Scatter plots show the association between the California Verbal Learning Test (CVLT-II) and the Framingham risk score (**a**), and total cholesterol levels (**b**); Box-and-whisker plot shows the association between CVLT-II and sex (**c**). Results are reported from a linear regression model (**a**) and a stepwise linear regression model (**b**,**c**).

Each point increase of the Framingham risk score corresponded to 0.21 lower score on CVLT-II (Coeff = −0.21; 95%CI = −0.39, −0.02) (Figure 1a). No associations were found between the Framingham risk score and the SDMT (Coeff = −0.07; 95%CI = −0.30, 0.15) and BVMT-R (Coeff = −0.16; 95%CI = −0.39, 0.07).

Looking at the Framingham risk score components, age was the only variable selected for the SDMT model (Coeff = −0.22; 95%CI = −0.45, 0.01). Male sex and higher total cholesterol levels corresponded to lower CVLT-II scores (Coeff = −8.54; 95%CI = −15.51, −1.57; and Coeff = −0.11; 95%CI = −0.20, −0.02, respectively) (Figure 1b,c), with smoking and hypertension also being selected for the CVLT-II model (Coeff = −5.31; 95%CI = −11.24, 0.60; and Coeff = −6.37;95%CI = −12.77, 0.02, respectively). Smoking and total cholesterol levels were selected for the BVMT-R model (Coeff = −0.07; 95%CI = −0.14, 0.01; and Coeff = −4.84; 95%CI = −9.72, 0.03, respectively).

When compared with patients with normal cerebral functional system score, no significant differences were found for patients scoring 1 (Coeff = 3.67; 95%CI = −2.48, 9.82) and ≥2 on the cerebral functional system (Coeff = 4.96; 95%CI = −1.23, 11.15).

## 4. Discussion

In our exploratory study, overall cardiovascular risk and, in particular, male sex and higher cholesterol levels were associated with worse verbal learning performance in MS. Among explored cognitive domains (and neuropsychological tests), verbal learning (CVLT-II) was the only being significantly associated with the Framingham risk score and its components, whilst attention and information processing speed (SDMT) and visuo-spatial function (BVMTR) were substantially unaffected by cardiovascular risk factors.

Cognitive dysfunction is known to be part of MS-related disability [25]. In line with this, we found that higher SDMT score was associated with higher EDSS and longer disease duration. SDMT had already shown to mirror MS disability progression, being associated with worse long-term disability, and higher risk of conversion to secondary progressive MS [25]. As such, the SDMT is considered the best screening test for cognitive impairment in MS, and could be integrated to standard clinical evaluation of MS patients [24]. Accordingly, we also found that the SDMT score was not associated with the Framingham risk score and its components, suggesting this test is not (or, at least, is less) affected by cardiovascular risk, reflecting, to a large extent, MS-specific cognitive changes [26].

Increasing evidence supports the association between cardiovascular risk factors and cognitive dysfunction in the general population. Cardiovascular risk factors drive atherosclerosis and ischemic lesions, with accelerated age-related brain volume loss [27], and can also directly increase amyloid deposition and plaque formation, with subsequent cognitive changes [28]. Recently, the impact of the cardiovascular risk factors on cognition has been demonstrated in midlife individuals (and, thus, on a population more similar to the MS epidemiology), with hypertension, diabetes, and smoking being associated with accelerated cognitive decline over 5 years [29]. Additionally, smoking was associated with earlier cerebral atrophy and cognitive decline, particularly involving flexibility and processing speed domains [18]. In our study, we focused on multiple cardiovascular risk factors and on their interaction, using the Framingham risk score. The CVLT-II was associated with the Framingham risk score and its components (male sex and total cholesterol levels), suggesting an impact of cerebrovascular damage on memory and verbal learning in MS. In line with this, the CVLT-II has been used to evaluate cognitive performances in patients with acute cerebrovascular disease, frequently showing learning and recall abnormalities [30]. Hence, the CVLT-II could be affected by cardiovascular variables, not only in the general population, but also in MS. Our results are possibly the consequence of chronic cardiovascular changes in MS, with reduced cerebral flow and subsequently impaired cognitive function [31]. The Framingham risk score calculates the risk of developing major cardiovascular events within 10 years, representing the global cardiovascular burden based on both modifiable and not modifiable risk factors. In our previous studies, the Framingham risk score was associated with MS relapses, disability, and DMT escalation, suggesting that cardiovascular comorbidity could affect the overall MS progression [11,21].

Among Framingham risk score components, male sex is a known negative prognostic factor in MS [1], as well as a non-modifiable cardiovascular risk factor [20]. As such, a number of gender-related hormonal, genetic, and environmental factors could mediate the association between male sex and low CLVT-II scores, with the contribution of both MS-related and cardiovascular-related cognitive changes [2,32]. An adverse lipid profile is a risk factor for worse MS prognosis, including higher volume and number of white-matter lesions on MRI, higher relapse rate, and disability progression [33,34]. Two previous studies also showed an association between lipid profile and frontal executive and global cognitive dysfunction in MS [35,36].

A number of limitations might have affected the present study, such as the cross-sectional design and the retrospective analysis of data, not allowing to demonstrate a causal association between the variables. Standard MRI acquisitions were unfortunately not available for the whole population, and, so, we were unable to assess the overall MS or cardiovascular burden on the brain. We used a relatively simple neuropsychological battery, only including three tests and, thus, not evaluating the full spectrum of cognitive changes. Another limitation could be the potential confounding effect of the different DMTs, that often profoundly influence the prognosis of MS patients; unfortunately, we could not consider this variable because of the small sample. Moreover, this study proposes a possible association of cardiovascular risk with one cognitive function (verbal learning) and lack of association to the others, but this exploratory finding should be thoroughly tested in a prospective setting, warranting further investigations in the long term.

## 5. Conclusions

In conclusion, cardiovascular risk was associated with cognitive impairment in MS. CVLT-II should be interpreted cautiously when assessing cognitive impairment in MS, especially in the presence of cardiovascular comorbidities. Lifestyle and pharmacological interventions on cardiovascular risk factors should be considered carefully in the management of MS, with possible effects also on cognitive function.

## Figures and Tables

**Table 1:** Demographic, clinical, cardiovascular, and neuropsychological variables.

	MS Patients (n *=* 69)
Age, years	48.63 ± 11.8
Sex, female n (%) EDSS, median (range)	49 (71%) 3.5 (1.0–8.0)
Relapsing-remitting MS, (%)	52 (75.36%)
Primary progressive MS, (%)	2 (2.9%)
Secondary progressive MS, (%)	15 (21.74%)
Disease duration, years	12.99 ± 8.11
Education, years	12.5 ± 3.77
Smokers, (%)	38 (55.07%)
Body Mass Index, kg/m^2^	25.22 ± 3.93
Systolic blood pressure, mmHg	138.8 ± 22.93
Anti-hypertensive treatment, (%)	30 (43.5%)
Diabetes, (%)	3 (4.35%)
Framingham risk score, %	11.9 ± 10.6
Symbol Digit Modalities Test, score	41.17 ± 11.59
California Verbal Learning Test II, score	37.88 ± 13.3
Brief Visuo-spatial Learning Test Revised, score	43.67 ± 10.53
Cerebral functional system score, (%) 0	
0	23 (33.3%)
1	23 (33.3%)
≥2	23 (33.3%)

## Data Availability

Data, material, and code are available upon request to the corresponding author.
